# Supplementary material for: Domestic carnivore interactions with wildlife in the Cape Horn Biosphere Reserve, Chile: husbandry and perceptions of impact from a community perspective
Source: PeerJ. 2018 Jan 4;6:e4124. doi: 10.7717/peerj.4124 (PMC5756619; doi:10.7717/peerj.4124)
Supplement: Supplemental Information 1 — Questionnaire for dog and cat owners, and non-owners in the Cape Horn Biosphere Reserve, translated from Spanish into English. Questions not relevant for non-owners were not asked. [file peerj-06-4124-s001.docx]

| *Pet demographic data*  Q1. How many dogs/cats do you have?  Q2. Please indicate for each of your dogs/cats: age, sex, origin, how it was obtained, for what purpose?  Q3. If you have females, please indicate: date of birth, no. of offspring, no. of dead offspring, no. of eliminated offspring, no. of given/sold offspring, where to?  Q4. Have any of your dogs/cats died during the last year? Yes/no. If yes, please indicate: age, sex, and cause.  Q5. Have you ever abandoned a cat/dog? Yes/no. If yes, please indicate: year, age, sex, and motivation.  Q6. Has there ever been a dog/cat eliminated in your family group? Yes/no. If yes, please indicate: year, age, sex, and motivation.  Q7. Have you given away or sold dogs/cats during the last year? Yes/no. If yes, please indicate: age, sex, and motivation.  Q8. Have you lost any dogs/cats during the last year? Yes/no. If yes, please indicate: for how long and possible explanation.  Q9. Have any of your dogs/cats ever got lost indefinitely? Yes/no. If yes, please indicate: year and possible explanation.  Q10. Would you prefer to have fewer or more dogs or do you have the right number? If you have fewer or more dogs than you prefer, please indicate why. |
| --- |
| *Pet care*  Q11. Are your dogs/cats spayed/neutered? If no, why not?  Q12. Are your dogs/cats treated against parasites? If yes, when was the last time?  Q13. Are your dogs/cats immunized against rabies? If yes, when was the last time?  Q14. What did your dogs/cats eat yesterday?  Q15. Which food type do you mainly feed your dogs/cats?  Q16. In how many households do your dogs/cats eat?  Q17. Do you feed street dogs? Yes/no. If yes, please indicate: how many, what kind of food, place, and motivation.  Q18. Do you contain or leash your dog? Yes/no. If yes, at day and/or at night?  Q19. If your dog is free-ranging, why is this?  Q20. What do you do with your dogs/cats when you have to travel?  Q21. What would you need to improve care of your dogs/cats? |
| *Attitudes*  Q22. What is your relationship with your dog? On a scale from 1 to 5, do you agree with the following sentences (1=do not agree, 5=completely agree)?  (a) I am happier because of my dog. (b) My dog is allowed in any part of my house. (c) My dog is part of my family. (d) My dog keeps me active. (e) I have modified my backyard for my dog.  Q23. Do you consider that there are disadvantages to having dogs/cats? Yes/no. If yes, what are they?  Q24. How do you perceive the dogs that you can see in the streets? On a scale from 1 to 5, do you agree with the following sentences (1=do not agree, 5=completely agree)?  (a) Street dogs enjoy their freedom. (b) Street dogs are dangerous to people. (c) Most street dogs do not have an owner. (d) The dogs are on the streets to better protect the houses. (e) Street dogs are a problem for tourism. (f) Street dogs do not roam into the forest. (g) Street dogs without owners should be eliminated. |
| *Personal experiences*  Q25. During the last year, did your dogs/cats come home with an animal/bird? Yes/no. If yes, which one?  Q26. During the last year, did you see your dogs/cats hunt another animal? Yes/no. When and which one?  Q27. Did you see dogs/cats outside the town during the last year? Yes/no. If yes, how many and where? Please show on the map. From where do these dogs come?  Q28. Did you see feral dog pups? Yes/no. If yes, please indicate: How many, age, when, and where.  Q29. Upon what do feral dogs feed?  Q30. Have you observed a problematic situation caused by dogs in town? Yes/no. If yes, which, when, and where? From where do these dogs come?  Q31. Have you observed a problematic situation caused by dogs outside town? Yes/no. If yes, which, when, and where? From where do these dogs come? |
| *Perception of impacts*  Q32. Do free-ranging dogs in town cause problems? Yes/no. If yes, which ones (up to three)?  Q33. In your opinion, what is a feral dog? Are there feral dogs on the island? Yes/no.  Q34. Do dogs/cats outside town cause problems? Yes/no. If yes, which ones (up to three)?  Q35. Do dogs outside town have negative impacts on wild animals and birds? Yes/no. If yes, which ones particularly?  Q36. To have fewer street dogs, what would be acceptable to do? |
| *Personal data*  Q37. Gender  Q38. Age  Q39. Highest completed education level: a. none, b. elementary school, c. high school, d. technical/vocational training, e. University  Q40. Do you permanently live in the Cape Horn County? How many years have you lived here?  Q41. How many persons live in your household? No. of adults, no. of children. |
| *Photo*  Q42. Do you give me permission to take a picture of your dogs/cats now? Yes/no. |
| *Only rural area*  Q43. Do you own livestock/sheep/pigs/chicken/etc.? Yes/no. If yes, which animals and how many?  Q44. Did you lose domestic animals because of dogs during this last year? If yes, please indicate: date, animal type, no. of dead animals, no. of injured animals, and possible origin of dog.  Q45. During the last 5 years, did you regularly (every year) lose animals? Yes/no.  Q46. What did you do to protect your animals against dogs?  Q47. What would be the best solution for you to protect your animals against dogs? |
